# LrrkA, a kinase with leucine‐rich repeats, links folate sensing with Kil2 activity and intracellular killing

**DOI:** 10.1111/cmi.13129

**Published:** 2019-11-07

**Authors:** Romain Bodinier, Jade Leiba, Ayman Sabra, Tania N. Jauslin, Otmane Lamrabet, Cyril Guilhen, Anna Marchetti, Yumi Iwade, Takefumi Kawata, Wanessa C. Lima, Pierre Cosson

**Affiliations:** ^1^ Department of Cell Physiology and Metabolism, Faculty of Medicine University of Geneva Geneva Switzerland; ^2^ Department of Biology, Faculty of Science Toho University Funabashi Japan

**Keywords:** *Dictyostelium*, DrkD, intracellular killing, Kil2, *Klebsiella pneumoniae*, LrrkA, magnesium

## Abstract

Phagocytic cells ingest bacteria by phagocytosis and kill them efficiently inside phagolysosomes. The molecular mechanisms involved in intracellular killing and their regulation are complex and still incompletely understood. *Dictyostelium discoideum* has been used as a model to discover and to study new gene products involved in intracellular killing of ingested bacteria. In this study, we performed random mutagenesis of *Dictyostelium* cells and isolated a mutant defective for growth on bacteria. This mutant is characterized by the genetic inactivation of the *lrrkA* gene, which encodes a protein with a kinase domain and leucine‐rich repeats. *LrrkA* knockout (KO) cells kill ingested *Klebsiella pneumoniae* bacteria inefficiently. This defect is not additive to the killing defect observed in *kil2* KO cells, suggesting that the function of Kil2 is partially controlled by LrrkA. Indeed, *lrrkA* KO cells exhibit a phenotype similar to that of *kil2* KO cells: Intraphagosomal proteolysis is inefficient, and both intraphagosomal killing and proteolysis are restored upon exogenous supplementation with magnesium ions. Bacterially secreted folate stimulates intracellular killing in *Dictyostelium* cells, but this stimulation is lost in cells with genetic inactivation of *kil2*, *lrrkA*, or *far1*. Together, these results indicate that the stimulation of intracellular killing by folate involves Far1 (the cell surface receptor for folate), LrrkA, and Kil2. This study is the first identification of a signalling pathway regulating intraphagosomal bacterial killing in *Dictyostelium* cells.

## 1 INTRODUCTION

Phagocytosis is used by both mammalian cells and environmental amoebae to ingest microorganisms, in particular bacteria. In mammals, one of the main functions of specialised phagocytic cells (e.g., neutrophils and macrophages) is to eliminate invading microorganisms and to protect the body against infections. Amoebae use phagocytosis to feed upon other microorganisms. In both mammalian cells and amoebae, intracellular destruction of ingested microorganisms is one of the first events following phagocytosis. To achieve this goal, ingested bacteria are rapidly transferred into acidic phagolysosomes, equipped to perform efficient killing. Phagocytosis, phagosome maturation, and intracellular bacterial killing are complex and interdependent processes involving multiple gene products. Consequently, our understanding of the molecular mechanisms ensuring intracellular killing is still largely incomplete (Dunn et al., 2017).

The *Dictyostelium discoideum* amoeba has been an instrumental model to study the molecular mechanisms controlling the dynamics of the actin cytoskeleton, phagocytosis, and intracellular killing of bacteria (Cosson & Soldati, 2008; Mori, Mode, & Pieters, 2018; Stuelten, Parent, & Montell, 2018). To the best of our current knowledge, molecular mechanisms involved in ingestion and killing of bacteria are largely similar in *Dictyostelium* and mammalian cells (Cosson & Soldati, 2008). Due to the relative ease with which haploid *Dictyostelium* cells can be grown, observed, and genetically altered, they have been largely used to discover and analyse the role of specific gene products in various facets of the phagocytic process. Identification of mutants with interesting phenotypic alterations has notably been a powerful method to discover new gene products involved in phagocytosis and intracellular killing.

One relatively easy way to identify *Dictyostelium* mutants with interesting phenotypic defects is to test their ability to grow in the presence of bacteria. Defects in various facets of phagocytosis (e.g., phagocytosis or intracellular bacterial killing) were indeed found to reduce the ability of *Dictyostelium* cells to feed upon various bacteria. This strategy has been successfully used to identify gene products involved in phagocytosis like SpdA (Dias et al., 2016) or in intracellular killing like Kil1 (Benghezal et al., 2006) Kil2 (Lelong et al., 2011) and Vps13F (Leiba et al., 2017). Importantly, growth in the presence of bacteria can be affected in many different manners, for example, by mutations decreasing the ability of the cell to recognise bacteria, to ingest them, to kill them, to digest them, or to make use of the nutrients. Defects in cellular motility, cell division, or gene expression could also modify the ability of a cell to grow in the presence of bacteria. As detailed in Section 3, from a practical point of view, this means that isolating mutants unable to grow in the presence of bacteria is a practical method to isolate interesting new mutants but a very poor method to characterize mutants.

In this study, we isolated and characterized a new *Dictyostelium* mutant unable to grow in the presence of Gram‐positive bacteria. The *lrrkA* (formerly *drkD*) gene, disrupted in this mutant, encodes a kinase with leucine‐rich repeats (LRRs). Our results reveal that LrrkA plays a key role in a pathway activating intracellular killing in response to folate.

## 2 RESULTS

### 2.1 *LrrkA* knockout cells are unable to feed upon Gram‐positive bacteria

In order to identify new gene products implicated in ingestion and killing of bacteria, we previously created a collection of random insertional mutants in a *kil2* knockout (KO) cell line (Leiba et al., 2017). We then tested the ability of individual clones to grow on a lawn of nonpathogenic strains of various bacterial species (*Klebsiella pneumoniae*, *Micrococcus luteus*, *Bacillus subtilis*, *Escherichia coli*, and *Pseudomonas aeruginosa*). We isolated in this manner a mutant clone unable to feed upon *M. luteus* and *B. subtilis*. We purified the genomic DNA of the mutant cells, digested it with ClaI, self‐ligated DNA fragments, and transformed them into competent bacteria. This led to the isolation of the inserted pSC plasmid with the genomic flanking regions (Figure S1a). Sequencing revealed that the mutagenic plasmid was inserted in the coding sequence of the *drkD* gene at position 1,782 (Figure S1a). As detailed below, this gene was renamed *lrrkA* in this study and in the dictyBase database.

In order to ascertain that the phenotype of the original mutant was due solely to the insertion of the plasmid in the *lrrkA* gene, we created deletion mutants where a part of the *lrrkA* gene was deleted. Specifically, we generated by homologous recombination three *lrrkA* KO clones as well as two *kil2–lrrkA* double KO clones (Figure S1b–d). These independent mutant clones were characterized in parallel and yielded indistinguishable phenotypes in all assays described below.

**Figure 1:**
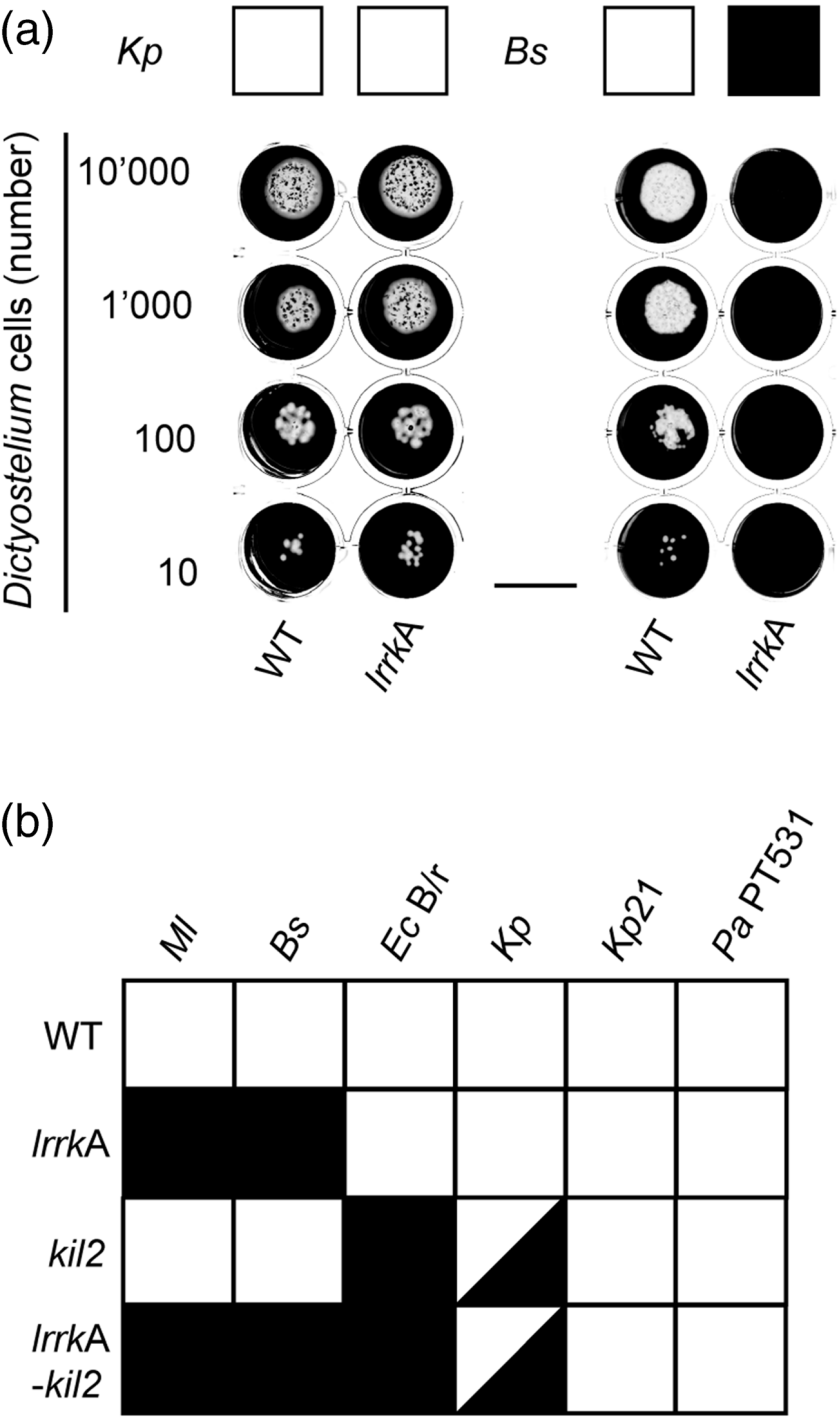
*lrrkA* knockout (KO) cells are unable to feed upon Gram‐positive bacteria. (a) To determine the ability of *Dictyostelium* cells to feed upon *Klebsiella pneumoniae* (*KpGe* strain*)*, *Dictyostelium* cells (10,000, 1,000, 100, or 10 cells) were deposited on a lawn of *KpGe*. After 4 days, wild‐type (WT) *Dictyostelium* cells created phagocytic plaques (white) in the bacterial lawn (black). *LrrkA* KO cells grew as well as WT cells on *K. pneumoniae* but were unable to grow on *Bacillus subtilis*. A white square indicates a growth comparable with that of WT cells, and a black square indicates a defective growth. (b) Several bacterial species were assessed as described above. *LrrkA* KO cells grew very poorly on both Gram‐positive bacteria tested (*Micrococcus luteus* and *B. subtilis*) but normally on other bacteria. *Bs*, *B. subtilis*; *Ec B/r*, *Escherichia coli B/r*; *Kp*, *K. pneumoniae KpGe*; *Kp*21, *K. pneumoniae* LM21; *Ml*, *M. luteus*; *Pa* PT531, *Pseudomonas aeruginosa* PT531. Scale bar: 1.5 cm

In order to precisely assess the ability of *Dictyostelium* cells to feed upon various bacteria, we deposited increasing numbers of cells (10 to 10,000) on a lawn of bacteria. After a few days, wild‐type (WT) *Dictyostelium* cells eliminated bacteria and created visible phagocytic plaques in bacterial lawns of *K. pneumoniae* and *B. subtilis* (Figure 1a). *LrrkA* KO cells grew as efficiently as WT cells on *K. pneumoniae*, but they were unable to feed upon *B. subtilis* (Figure 1a). We also assessed growth of *lrrkA* KO cells on a wider range of bacteria (*M. luteus*, a Kp21 *K. pneumoniae* strain, *E. coli*, and one nonpathogenic *P. aeruginosa* strain), and this analysis revealed that *lrrkA* KO cells were unable to feed upon the two Gram‐positive bacteria tested but showed no defect when grown on a lawn of Gram‐negative bacteria (Figure 1b).

### 2.2 LrrkA is a kinase with LRRs and no Roc GTPase domain

**Figure 2:**
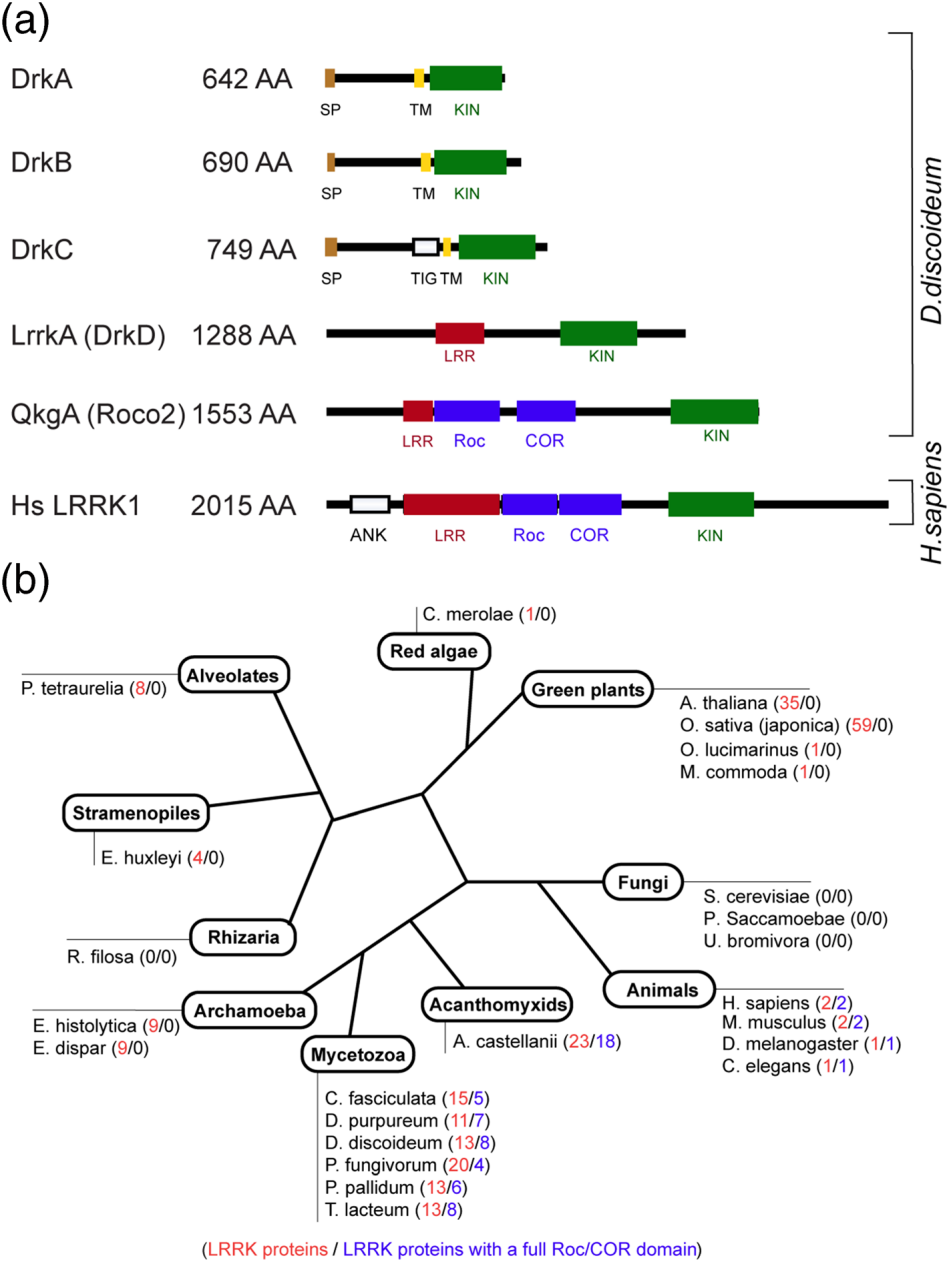
DrkD/LrrkA belongs to the family of leucine‐rich repeat (LRR) kinases. (a) Organisation of functional domains of DrkA–DrkD, of the *Dictyostelium* ROCO2 kinase, and of the human LRR kinase 1. DrkA, DrkB, and DrkC contain a putative signal peptide and a putative transmembrane domain situated N‐terminally of the kinase domain. LrrkA/DrkD, Roco 2, and human LRRK1 contain LRRs and a kinase domain. In addition, ROCO proteins (Roco 2 and human LRRK1) contain a Roc and a Cor domain. (b) Schematic phylogenetic tree representing the number of LRR kinases (with or without a Roc/COR domain) in eukaryotes (indicated in red) and the number of LRR kinases containing a full Roc/COR domain (indicated in blue)

The *lrrkA* gene analysed in this study was originally named *drkD*. The Drk family as originally defined contained four kinases characterized by the presence of a conserved putative kinase domain (Araki et al., 1998). However, there are notable differences between DrkA, DrkB, and DrkC on one side and DrkD on the other side. First, DrkA, DrkB, and DrkC have a signal peptide and a transmembrane domain, both absent in DrkD (Figure 2a). Second, DrkD contains seven LRRs, not found in DrkA, DrkB, and DrkC (Figure 2a). Third, the size of DrkD (1,288 residues) differs widely from that of DrkA, DrkB, and DrkC (642–749 residues; Figure 2a). Consequently, we propose to move DrkD out of the Drk family and to rename it LrrkA (LRR kinase).

Cytosolic kinases containing LRRs are found in a variety of species over the whole evolutionary tree (Figure 2b). There are, for example, eight LRR‐containing kinases in the unicellular ciliate *Paramecium tetraurelia* and 35 in the plant *Arabidopsis thaliana*. Although the phylogenetic relationships remain to be determined, a variation on this basic structure appeared after the divergence of plants: Starting at this point, some LRR‐containing kinases contain in addition a Roc/COR GTPase domain, and they are often referred to as Roco kinases. In Mycetozoa (including *D. discoideum*) and Acanthomyxids (*Acanthamoeba castellanii*), many LRR‐containing kinases can be found, some of which belong to the Roco subfamily (Figure 2b). In animals, LRR‐containing kinases are less numerous, and they all belong to the Roco subfamily. In human, there are only two Roco kinases, named LRRK1 and LRRK2 (Figures 2b and S2). We did not identify LRR‐containing kinases in fungi (*Saccharomyces cerevisiae*, *Paramicrosporidium saccamoebae*, and *Ustilago bromivora*; Figure 2b). A more complete description of Roco proteins, including proteins devoid of a kinase domain, was recently published (Wauters, Versees, & Kortholt, 2019).

Note that the classification of LRR kinases and Roco kinases is not devoid of some ambiguity, because Roco kinases can contain incomplete Roc/COR domains, or be devoid of LRRs. In *Dictyostelium*, we identified a total of 13 LRR kinases (Figure S2):

- LrrkA.
- DDB_G0278509 has an overall structure similar to that of LrrkA. Primary sequence analysis revealed one kinase domain, 13 LRRs, and no other functional domains. We consequently named it LrrkB.
- DDB_G0278909 contains one kinase domain, six LRRs, and 13 HEAT repeats. The kinase domain misses a catalytic aspartate and is expected to be inactive.
- Eight Roco kinases contain a full Roc/COR domain and LRRs (Roco 1–6, 8, and 11).
- Two kinases classified as Roco kinases (Roco 9 and 10) contain LRRs but an incomplete or absent Roc/COR domain.
- Roco 7 has been classified as a Roco kinase. It contains an incomplete Roc/COR domain and no LRRs and is thus strictly speaking not an LRR kinase.

In order to verify that LrrkA has a functional kinase domain, we expressed myc‐tagged LrrkA, both WT, and a catalytically inactive version (K877A). After immunoprecipitation of LrrkA–myc with an anti‐myc antibody, anti‐phosphoserine antibodies revealed that a serine is phosphorylated in LrrkA but not in the inactive mutant (Figure S3). No tyrosine phosphorylation was detected with an anti‐phosphotyrosine antibody. These results suggest that LrrkA is a serine (and presumably a serine/threonine) kinase and that it is capable of autophosphorylation.

### 2.3 The structure and pH of phagocytic compartments is unaffected in *lrrkA* KO cells

**Figure 3:**
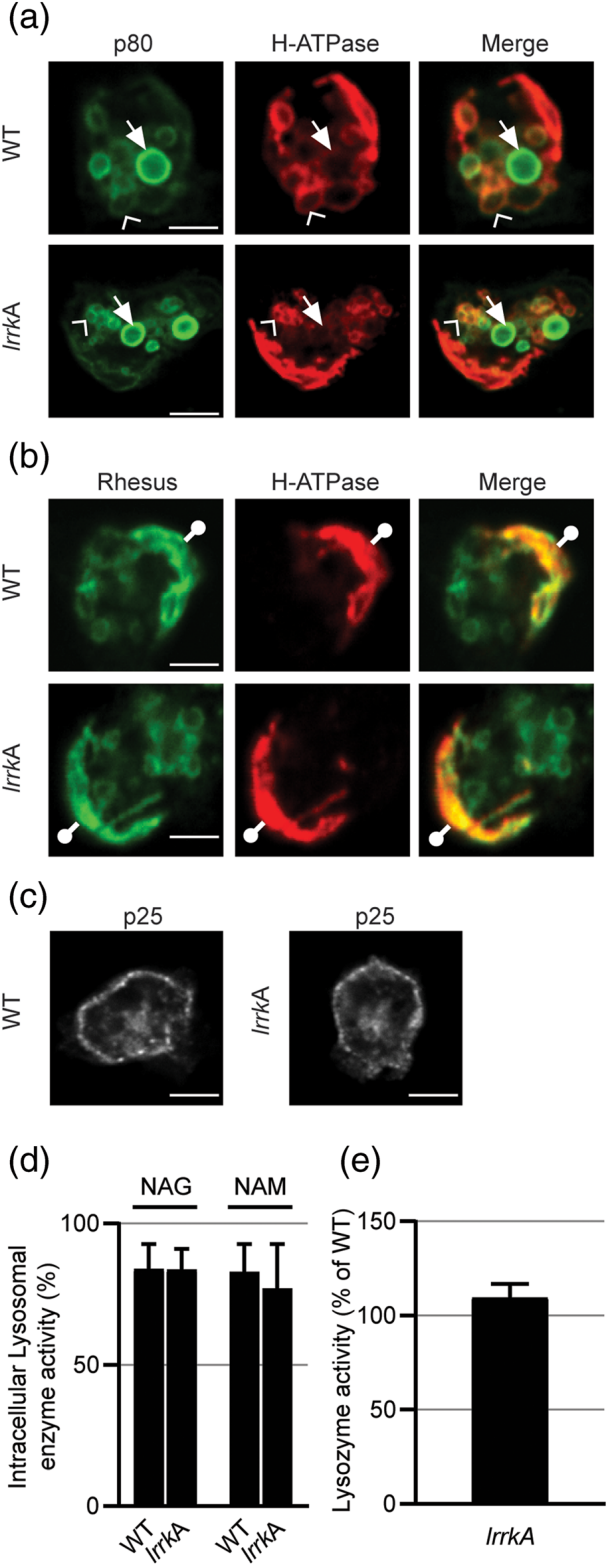
General organisation of cellular compartments is similar in *lrrkA* knockout (KO) and in wild‐type (WT) cells. (a) Immunofluorescence labelling of p80 and H^+^‐ATPase allowed to identify lysosomes (arrowheads; p80^+^ and H^+^‐ATPase^+^) and post‐lysosomes (arrows; p80^+^ and H^+^‐ATPase^−^). (b) Immunofluorescence labelling of rhesus and H^+^‐ATPase allowed to identify contractile vacuole (circles with bar; rhesus^+^ and H^+^‐ATPase^+^). (c) Immunofluorescence labelling of recycling endosomes enriched in p25. Scale bar in (a–c): 5 μm. (d) Intracellular retention of lysosomal glycosidases is as efficient in WT as in *lrrkA* KO cells. After 4 days of culture in HL5 medium, *Dictyostelium* cells were recovered by centrifugation, and the activity of two lysosomal enzymes (NAG, *N*‐acetyl‐β‐glucosaminidase; NAM, α‐mannosidase) was measured in cell pellets and in supernatants using chromogenic substrates. The percentage of intracellular glycosidase activity was not different for WT and *lrrkA* KO cells (mean ± standard error of the mean, *N* = 7, paired Student's *t* test, NAG *p* = .948, NAM *p* = .492). (e) Intracellular levels of lysozyme activity are similar in WT and *lrrkA* KO cells (mean ± standard error of the mean, paired Student's *t* test, *N* = 5, *p* = .251)

Many mutants showing a reduced ability to feed on bacteria exhibit a defect in the structure or function of the endocytic or phagocytic pathways. Consequently, we first checked whether the basic structure of the phagocytic pathway was perturbed in *lrrkA* KO cells. For this, we visualised the structure of the endocytic pathway by immunofluorescence, using antibodies against the main endocytic compartments. The structure of the contractile vacuole, visualised with an anti‐rhesus antibody (Benghezal, Gotthardt, Cornillon, & Cosson, 2001), of recycling endosomes (p25 positive; Charette, Mercanti, Letourneur, Bennett, & Cosson, 2006), of lysosomes (p80 positive and H^+^‐ATPase positive; Ravanel et al., 2001), and of post‐lysosomes (p80 high and H^+^‐ATPase negative; Ravanel et al., 2001) was indistinguishable in *lrrkA* KO and in WT cells (Figure 3a–c).

We next assessed the intracellular retention of two lysosomal glycosidases (α‐mannosidase and *N*‐acetyl‐glucosaminidase) and found no difference between WT and *lrrkA* KO cells (Figure 3d), suggesting that lysosomal targeting is not grossly deficient in *lrrkA* KO cells. Finally, the activity of intracellular lysozymes, hydrolytic enzymes potentially implicated in intracellular killing in phagocytic cells (Muller et al., 2005), was indistinguishable in WT and in *lrrkA* KO cells (Figure 3e).

**Figure 4:**
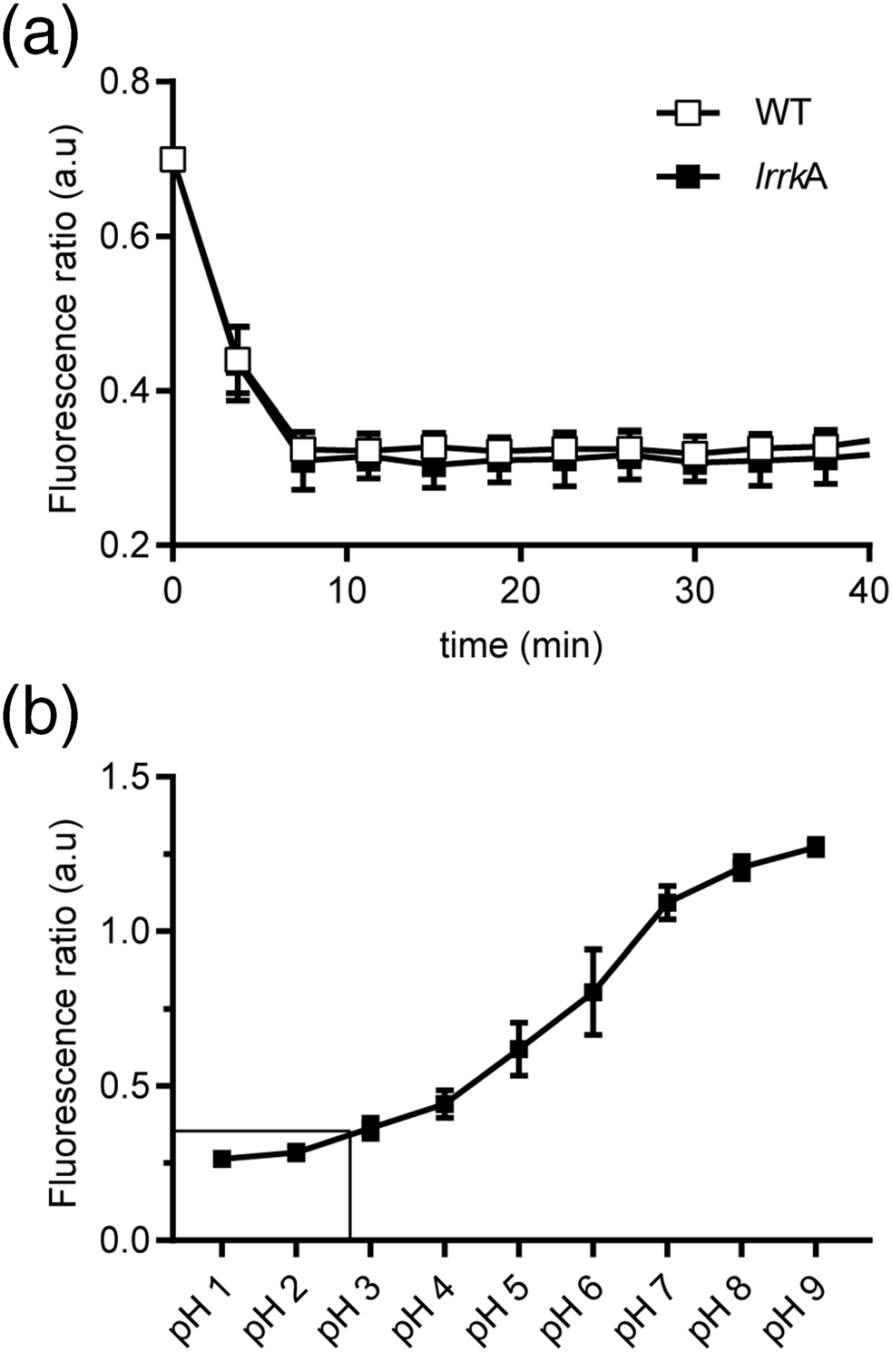
Acidification of phagosomes is unaffected in *lrrkA* knockout (KO) cells. (a) Wild‐type (WT) and *lrrkA* KO cells were allowed to internalise 3‐μm‐diameter beads coated with two fluorophores (pH‐sensitive FITC and pH‐insensitive Alexa 594) and imaged every 75 s for 3 hr. The pH was deduced from the fluorescence 488/594 nm ratio (mean ± standard error of the mean, *N* = 4, *n* = 40 beads for each strain). The kinetics of acidification and the pH value in phagolysosomes were identical in *lrrkA* KO and in WT cells. The fluorescence ratio in acidic phagolysosomes is 0.324. (b) Calibration curve of the pH‐sensitive beads in buffer ranging from pH 1 to 9. The dynamic range allows to discriminate variations of pH from 2 to 8 (mean ± standard error of the mean, *N* = 3, *n* = 100 beads per pH). A fluorescence ratio of 0.324 corresponds to a pH value between 2.5 and 3

We then measured the pH of phagosomes after ingestion of beads coupled to two fluorophores, one of which (FITC) is extinguished at low pH, whereas the other one (Alexa Dextran 594) is not. In WT cells, a very rapid acidification was observed after formation of the phagosome (Figure 4a), and this very acidic pH was maintained for more than 30 min, that is, largely after intracellular killing was completed. Interestingly, a calibration curve of fluorescence extinction at low pH indicated that FITC coupled to beads was quenched gradually between pH 2 and 8 (Figure 4b). This behaviour differs from that of FITC coupled to a soluble dextran, which is completely quenched at pH 5 or below (Marchetti, Lelong, & Cosson, 2009). This allows us to extrapolate that the pH in phagosomes may be as low as 2.5, in agreement with previous results suggesting that *Dictyostelium* lysosomes are exceptionally acidic (inferior to 3.5; Marchetti et al., 2009). In *lrrkA* KO cells, acidification kinetics were indistinguishable from those in WT cells (Figure 4a), revealing that at least in beads‐containing phagosomes, acidification is not perturbed in *lrrkA* KO cells.

In summary, immunofluorescence analysis and pH measurement did not reveal major defects in the structure and organisation of the endocytic pathway in *lrrkA* KO cells.

### 2.4 *LrrkA* KO cells are defective for intracellular killing of *K. pneumoniae* bacteria

**Figure 5:**
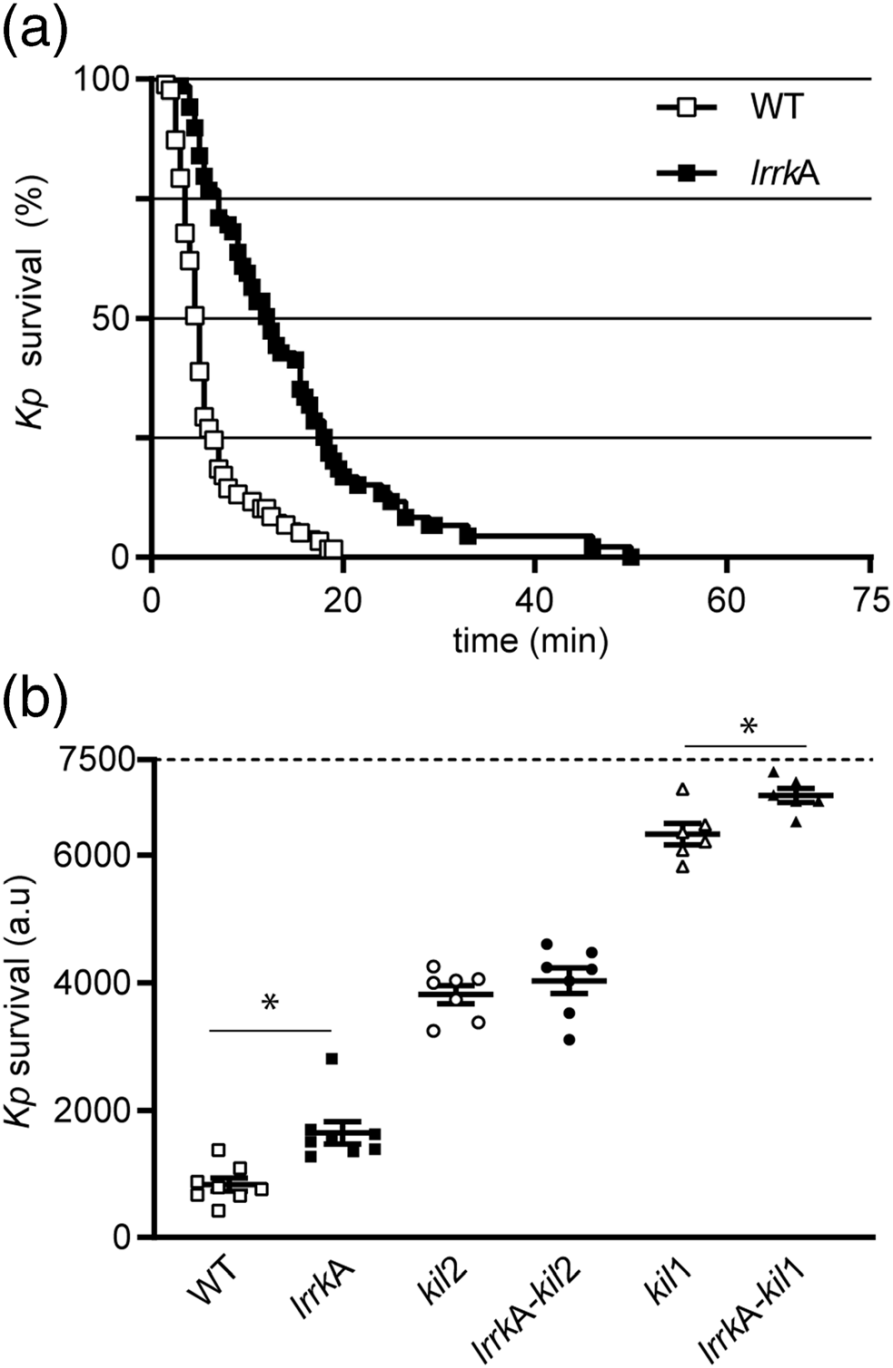
Intracellular killing of *Klebsiella pneumoniae* is impaired in *lrrkA* knockout (KO) cells. *Dictyostelium* cells were incubated with GFP‐expressing *K. pneumoniae* (Kp‐GFP) in phosphate buffer–sorbitol for 2 hr. Cells were observed by phase‐contrast and fluorescence microscopy, and the ingestion and intracellular killing of Kp‐GFP were monitored. (a) The probability of bacterial survival following ingestion is represented as a Kaplan–Meyer estimator for one experiment in wild‐type (WT) cells (white squares) and *lrrkA* KO cells (black squares). (b) For each experiment, the survival of bacteria was determined by measuring the area under the survival curve from 0 to 75 min. Each dot is the result of a separate experiment. Intracellular killing was significantly slower in *lrrkA* KO cells and in *kil2* KO cells than in WT cells (*p* = .013; paired Student's *t* test, *N* = 8 independent experiments). Intracellular killing was not slower in *lrrkA–kil2* KO cells than in *kil2* KO cells (*p* = .510; paired Student's *t* test, *N* = 7 independent experiments), but it was significantly slower in *lrrkA–kil1* KO cells than in *kil1* KO cells (*p* = 4.10^−4^; paired Student's *t* test, *N* = 6 independent experiments). Total number of events observed: WT = 351, *lrrkA* = 496, *kil2* = 274, *lrrkA–kil2* = 252, *kil1* = 120, and *lrrkA–kil1* = 120

The fact that *lrrkA* KO cells grow poorly in the presence of some bacteria may be due to their inability to kill efficiently ingested bacteria. In order to test this hypothesis, cells were allowed to ingest GFP‐expressing *K. pneumoniae* bacteria, and the survival of the bacteria was monitored as previously described (Leiba et al., 2017). Previous experiments have established that loss of fluorescence accompanies loss of bacterial viability (Lelong et al., 2011). Using this assay, WT cells were found to kill ingested *K. pneumoniae* shortly after phagocytosis (Figure 5a; median killing time 6 min). In *lrrkA* KO cells, intracellular killing was significantly slower (Figure 5a; median killing time 14 min). This delay in intracellular killing was highly reproducible in multiple experiments (Figure 5b). As previously described (Lelong et al., 2011), intracellular killing was also delayed in *kil2* KO cells (Figure 5b). Remarkably, the effect of the two mutations was not additive: Killing was not slower in *kil2–lrrkA* double KO cells than in *kil2* KO cells (Figure 5b). This observation suggests that Kil2 and LrrkA play partially redundant roles in intracellular killing. On the contrary, *kil1–lrrkA* double KO cells killed bacteria even slower than *kil1* KO cells (Figure 5b), suggesting that Kil1 and LrrkA do not play redundant roles in intracellular killing.

We also tested in a similar manner the ability of *lrrkA* KO cells to kill ingested *B. subtilis* expressing a fluorescent mCherry. Interestingly, *lrrkA* KO cells killed these Gram‐positive bacteria as efficiently as WT cells (Figure S4). This result indicates that different killing mechanisms are involved in the killing of *K. pneumoniae* and *B. subtilis*. It is at first glance surprising that *lrrkA* KO cells are unable to grow in the presence of Gram‐positive bacteria, although they can kill them normally. Similarly, it is surprising that *lrrkA* KO cells grow normally in the presence of *K. pneumoniae* bacteria, while they kill them inefficiently. This lack of congruence between these two assays is discussed in Section 3.

### 2.5 Phagosomal proteolysis is defective in *lrrkA* KO cells

**Figure 6:**
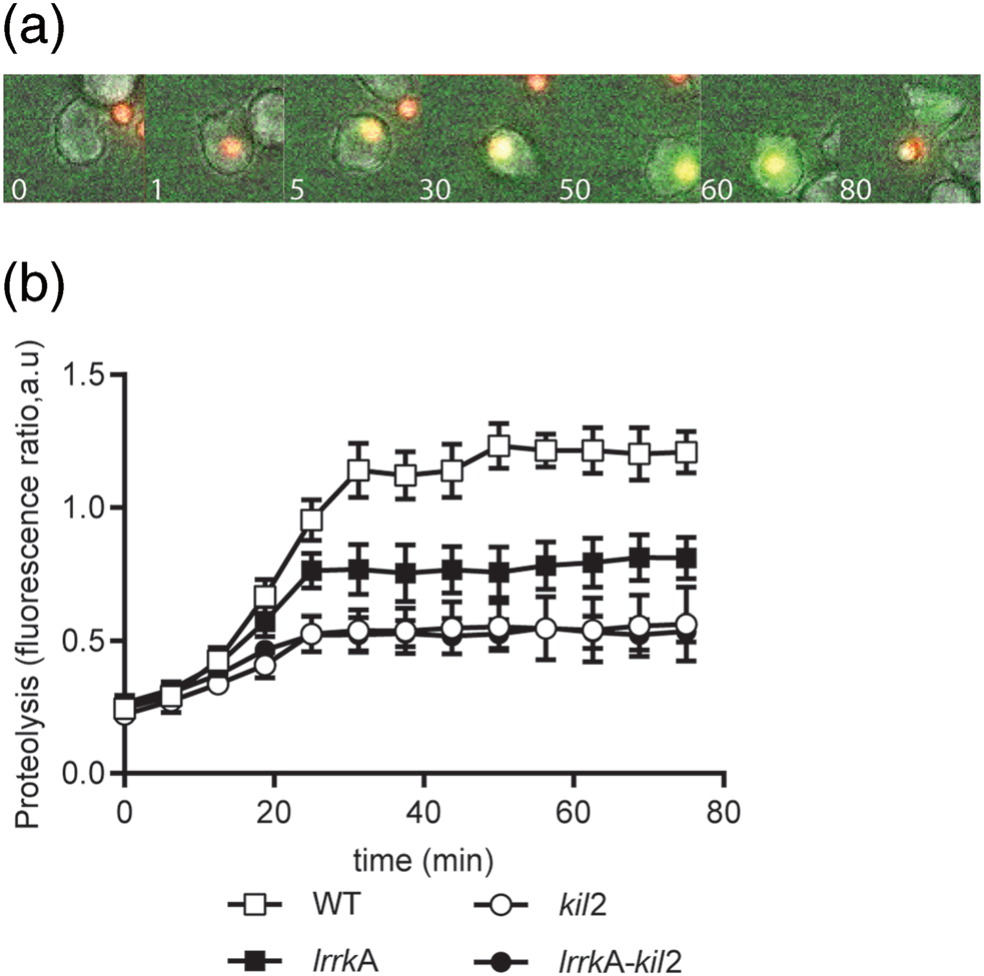
Proteolytic activity in phagosomes is reduced in *lrrkA* knockout (KO) cells. (a) Cells were allowed to internalise beads coated with a quenched fluorophore (DQ™ Green BSA) and a proteolysis‐insensitive dye (Alexa 594) and were imaged every 75 s. Representative, successive pictures of a wild‐type (WT) cell ingesting a bead and processing it over 80 min are shown. Upon degradation of BSA by proteases, the DQ™ Green is released in phagosomes, and the corresponding fluorescence increases. (b) To quantify proteolysis, we plotted the 488/594‐nm fluorescence ratio as a function of time following phagocytosis. In all cells, DQ green fluorescence reached a plateau approximately 40 min after phagocytosis. The fluorescence ratio was lower in *lrrkA* KO cells than in WT cells and even lower in *kil2* KO cells or in *kil2–lrrkA* KO cells (mean ± standard error of the mean, *N* = 4, *n* = 40 beads for each strain)

Previous experiments have shown that the killing defect observed in *kil2* KO cells is accompanied by a defective activity of proteases in phagosomes (Lelong et al., 2011). If LrrkA and Kil2 do play redundant roles, a similar phenotype may be expected in *lrrkA* KO cells. In order to test the activity of proteases in phagosomes, cells were allowed to ingest silica beads coated with Alexa Fluor 594 and DQ Green‐labelled BSA at a self‐quenching concentration. Proteolysis releases DQ Green from the beads and increases its fluorescence (Sattler, Monroy, & Soldati, 2013; Figure 6a). Quantification of the images obtained by fluorescence microscopy confirmed that phagosomal proteolysis was less efficient in *kil2* KO cells than in WT cells (Figure 6b).

Proteolysis was also less efficient in *lrrkA* KO cells than in WT cells, although the defect was less pronounced than in *kil2* KO cells (Figure 6b). Proteolysis was however not slower in *kil2–lrrkA* double KO cells than in *kil2* KO cells (Figure 6b). These results suggest that Kil2 and LrrkA play partially redundant roles in controlling proteolysis in *Dictyostelium* phagosomes.

### 2.6 Magnesium supplementation alleviates the killing and proteolysis defects of *lrrkA* KO cells

**Figure 7:**
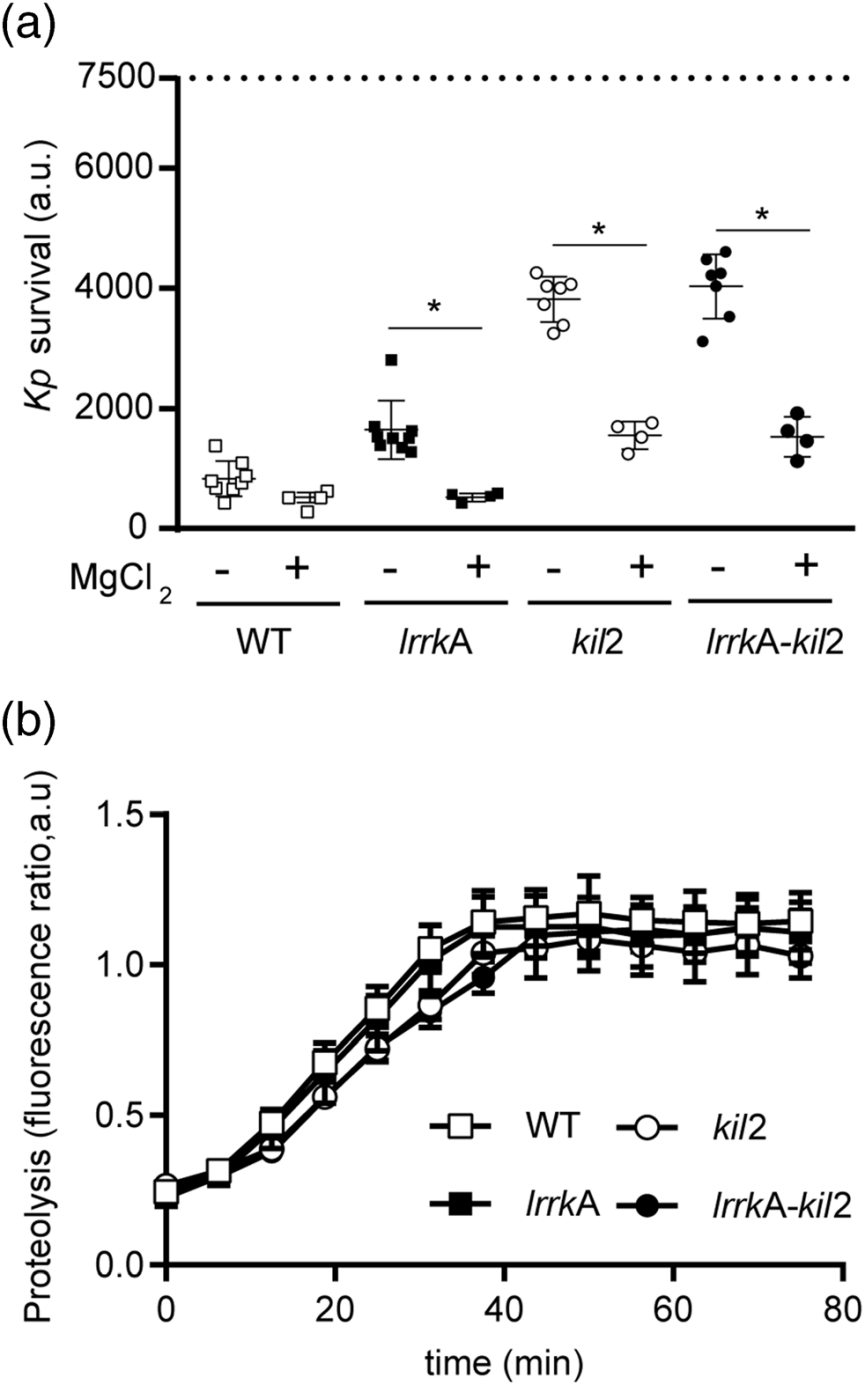
Exogenous addition of magnesium restores intracellular killing and proteolytic activity in *lrrkA* knockout (KO), *kil2* KO, and *lrrkA–kil2* KO cells. (a) Intracellular killing of *Klebsiella pneumoniae* was determined in wild‐type (WT), *lrrkA* KO, *kil*2 KO, and *lrrkA*–*kil*2 KO cells in the presence or absence of MgCl_2_ (1 mM), as described in Figure 5. In these experiments, MgCl_2_ was directly added in the medium containing cells and bacteria. Exogenous MgCl_2_ accelerated significantly intracellular killing in all mutant cells (*p* < 10^−4^; paired Student's *t* test, *N* = 5 independent experiments). (b) Proteolytic activity was measured in phagosomes in the presence of 1 mM of MgCl_2_ as detailed in Figure 6. Normal levels of phagosomal proteolytic activity were restored in all mutant cells by addition of exogeneous magnesium

It has been previously proposed that Kil2 stimulates intraphagosomal proteolysis by pumping magnesium ions into the phagosome. This proposal is mainly based on the observation that exogenous addition of magnesium restores normal intraphagosomal killing and proteolysis in *kil2* KO cells (Lelong et al., 2011). We consequently determined the effect of exogenous magnesium on intracellular killing and proteolysis in WT, *kil2*, and *lrrkA* KO cells (Figure 7). Addition of exogenous magnesium restored efficient killing in *lrrkA* KO cells (Figure 7a). As previously reported, exogenous magnesium also greatly stimulated killing in *kil2* KO cells. An identical effect was observed in *kil2–lrrkA* double KO cells (Figure 7a).

Exogenous magnesium also restored normal phagosomal proteolysis in *lrrkA* KO cells, *kil2* KO cells, and *lrrkA–kil2* double KO cells (Figure 7b). These results further suggest that LrrkA and Kil2 play redundant roles in the control of magnesium‐dependent phagosomal proteolysis and bacterial killing.

### 2.7 Stimulation of intracellular killing by folate requires Far1, LrrkA, and Kil2

**Figure 8:**
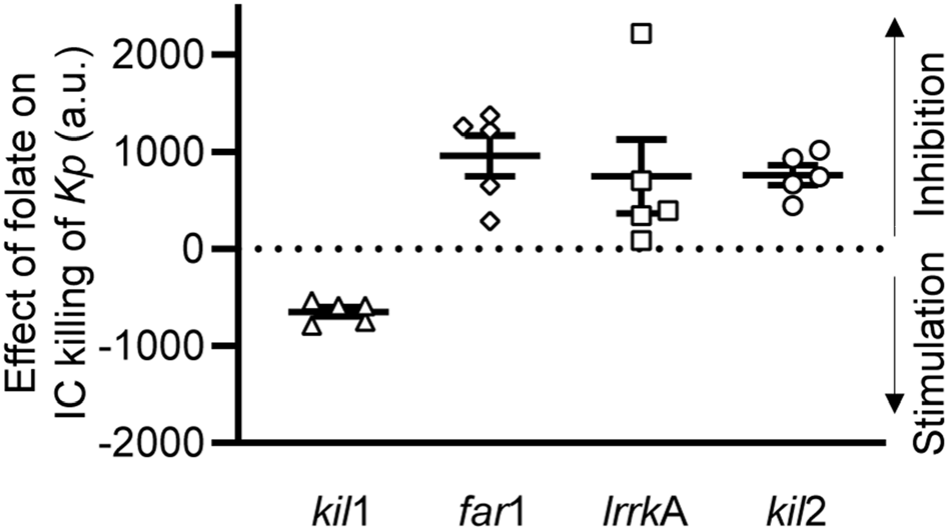
Far1, LrrkA, and Kil2 participate in a folate‐sensitive pathway stimulating intracellular killing. Intracellular (IC) killing of *Klebsiella pneumoniae* (Kp) was determined in *far1* knockout (KO), *lrrkA* KO, *kil*2 KO, and *kil1* KO cells in the presence or absence of folate (1 mM), as described in Figure 5 (*N* = 5). Each dot represents the difference of area under the curve for each strain with or without folate. All values above zero indicate that the addition of folate slowed intracellular killing. Conversely a value below zero indicates that addition of folate accelerated intracellular killing. Folate stimulated intracellular killing in *kil1* KO cells but not in *far1* KO, *lrrkA* KO, and *kil2* KO cells, revealing the role of Far1, LrrkA, and Kil2 in increasing killing upon folate sensing

The results presented above suggest that LrrkA can modulate Kil2 activity and intraphagosomal killing. Little is known about the regulation of intracellular killing mechanisms in *Dictyostelium* in response to extracellular cues. We recently reported that folate, which is synthesized and secreted by many bacteria, can stimulate killing (Leiba et al., 2017). This effect was initially detected in killing‐deficient *vps13F* KO cells: Upon exposure to folate, efficient killing was restored in *vps13F* KO cells (Leiba et al., 2017). Our previous results have also shown that folate does not stimulate intracellular killing in *kil2* KO cells (Leiba et al., 2017; Figure 8), suggesting that Kil2 may be necessary for the response to folate. If LrrkA stimulates Kil2 activity, it may represent a missing link between folate sensing and Kil2 activity. To test this hypothesis, we measured the ability of folate to stimulate intracellular killing of ingested *K. pneumoniae* in various mutant cells.

**Figure 9:**
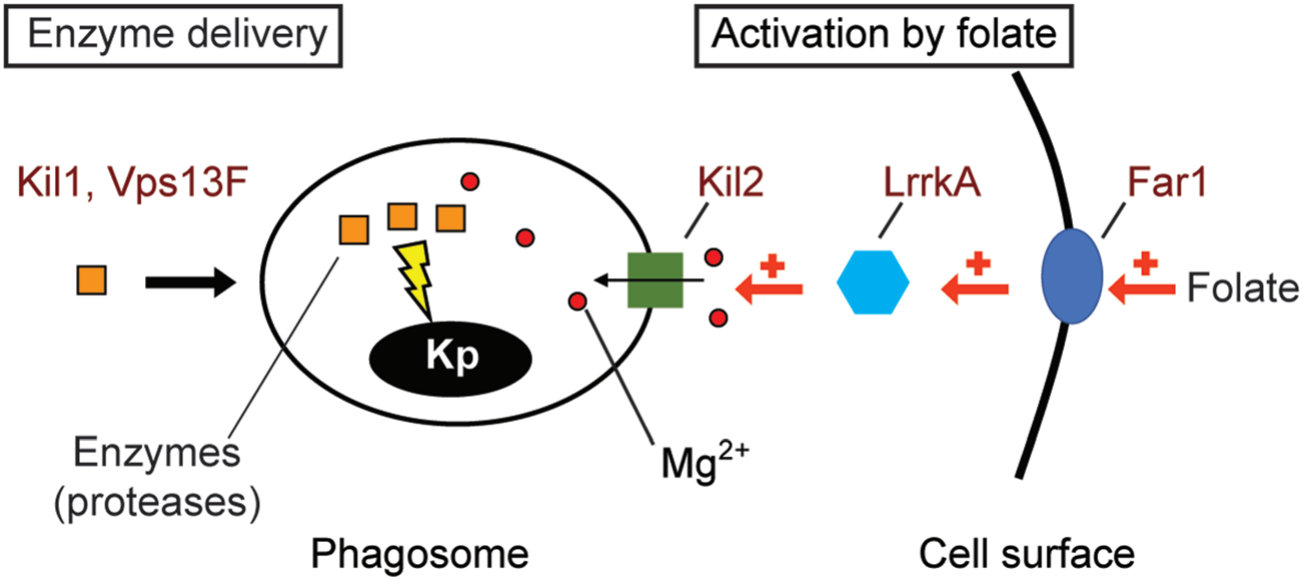
Intracellular killing of *Klebsiella pneumoniae*. This simple scheme describes our working model incorporating the results described in this study. Folate activates Far1, which activates LrrkA, resulting in the stimulation of the activity of Kil2 and transfer of magnesium ions (Mg^2+^) from the cytosol to the phagosomal lumen. In the presence of increased levels of magnesium ions, lysosomal enzymes kill more efficiently ingested *K. pneumoniae* bacteria (Kp). Kil1 and Vps13F play a distinct role in the delivery of properly modified enzymes (notably proteases) to phagosomes

As previously reported for *vps13F* KO cells, folate stimulated intracellular killing in *kil1* KO cells (Figure 8). Remarkably, intracellular killing was not stimulated (and actually inhibited) in *lrrkA* KO cells upon exposure to folate (Figure 8). Far1 has been identified as the main folate receptor at the cell surface (Pan, Xu, Chen, & Jin, 2016), and intracellular killing was also not stimulated by folate in *far1* KO cells (Figure 8). Taken together, these results reveal the existence of a signalling pathway where folate is recognised at the cell surface by the Far1 receptor, leading to the sequential activation of LrrkA and Kil2 and ultimately to the activation of intraphagosomal killing of bacteria (Figure 9).

## 3 DISCUSSION

In this study, we identified a new molecular mechanism controlling intracellular killing of *K. pneumoniae*. LrrkA is a kinase with LRRs, and its genetic ablation results in a markedly slower intracellular killing of ingested *K. pneumoniae*, although the overall structure of the phagocytic pathway is not defective in these cells. Genetic analysis of *kil2–lrrkA* double KO cells, as well as analysis of the phenotype of *lrrkA* KO cells indicate that LrrkA stimulates the activity of Kil2, a putative magnesium transporter in the phagosomal membrane. Magnesium is essential for optimal activity of phagosomal proteases and for efficient killing of ingested *K. pneumoniae*. In addition, our results indicate that LrrkA links the sensing of bacterially secreted folate to the activity of Kil2 and intracellular killing. A schematic description of the roles of folate, LrrkA, Kil2, magnesium ions, and proteases in intraphagosomal killing of bacteria is depicted in Figure 9. It is logical to assume that *Dictyostelium* uses bacterial‐sensing mechanisms to adapt intraphagosomal killing to the ingested bacteria, but to our knowledge, this is the first study suggesting the existence of a folate‐sensing pathway stimulating intracellular killing.

In order to propose a unified model, we have been led to revisit the interpretation of previously published results (Leiba et al., 2017). According to our current interpretation, the fact that intracellular killing is stimulated by folate in *vps13F* KO cells but not in *kil2* KO cells indicates that Vps13F is not involved in stimulation of intracellular killing by folate, whereas Kil2 is. Similarly, Far1 and LrrkA are essential for the stimulation of intracellular killing by folate, whereas Kil1 is not. Moreover, the fact that *kil2–vps13F* double KO cells are more defective in intracellular killing than *kil2* KO cells indicates that the function of Kil2 is not dependent on Vps13F. According to this new interpretation, we suggest that Kil1 and Vps13F play a role in intracellular killing distinct from that of Far1, Kil2, and LrrkA: while the folate–Far1–LrrkA–Kil2 pathway controls ionic composition of phagosomes, Kil1 and Vps13F may play a role in the modification and phagosomal targeting of lysosomal enzymes involved in killing (Figure 9). Although this model accounts for the results presented in this study, we are fully aware that the proposed scheme is overly simple and that it will most probably be refined by further studies.

Screening for mutants exhibiting defective growth in the presence of bacteria has proven a powerful method to identify mutants defective in intracellular killing, but we would like to point out the difficulty in interpreting precisely the results of these growth assays. For example, in the current study, *lrrkA* KO cells were shown to grow efficiently on a lawn of *K. pneumoniae* but not on a lawn of *B. subtilis*. Simple logic would lead to predict that *lrrkA* KO cells kill *K. pneumoniae* efficiently and *B. subtilis* inefficiently. The opposite result is reported in this study. This is not the first case of a discrepancy between assays measuring different facets of *Dictyostelium* physiology: For example, *kil1* KO cells kill *K. pneumoniae* very poorly, yet they grow almost as efficiently as WT cells on a lawn of *K. pneumoniae* (Le Coadic et al., 2013). These results stress the fact that these two assays (growth in the presence of bacteria and killing of bacteria) measure different parameters in different situations. To measure killing, we use cells growing exponentially in rich liquid medium and measure over a short time (2 hr maximum) their ability to kill ingested bacteria. On the contrary, growth in the presence of bacteria is assessed over a period of 5 to 10 days. During that time, *Dictyostelium* cells must achieve numerous functions to be able to grow: sense, ingest, kill, and digest bacteria and also migrate on the plate, grow, divide, and so forth. Any alteration in one or several of these functions can potentially alter in a highly unpredictable way the final outcome. In addition, previous studies have shown that the pattern of gene expression is profoundly different in *Dictyostelium* cells growing in HL5 medium and on a lawn of bacteria and also differs depending on the bacterial species forming the lawn (Nasser et al., 2013). Consequently, the relative importance of any given gene product may vary significantly between these different conditions. To obtain meaningful comparisons, we have chosen to focus on a standard invariant growth condition, that is, cells growing in HL5 medium. It may be of interest in future studies to establish if the mechanisms allowing efficient killing are significantly modulated when *Dictyostelium* cells are grown continuously in the presence of bacteria. This may allow to reconcile the results obtained in growth assays with those obtained during more precise characterization of *Dictyostelium* mutants. From a practical perspective, the fact that a mutant cell is not capable of feeding upon given bacteria is often indicative of a defect in the structure or function of the phagocytic pathway. It cannot however be equated with specific alterations in cell physiology, such as an inability to kill specific bacteria. Specific assays must be used to measure alterations in various facets of *Dictyostelium* physiology.

Finally, this study reveals a high degree of specificity in the mechanisms controlling intracellular killing of different bacteria: Killing of *K. pneumoniae* is slower in *lrrkA* KO cells than in WT cells, but killing of *B. subtilis* is not affected. Similar results were reported previously when the phenotype of *kil1* and *kil2* KO mutants was analysed (Benghezal et al., 2006; Lelong et al., 2011). Overall, our current knowledge strongly suggests that *Dictyostelium* uses vastly different mechanisms to kill different types of bacteria. Although our knowledge of the intracellular mechanisms controlling intracellular killing of *K. pneumoniae* is growing, the mechanisms controlling intracellular killing of other bacteria remain to be identified.

It is not easy to compare the function of *Dictyostelium* LrrkA with human Lrr kinases, because the family of Lrr kinases is more diverse in *Dictyostelium* than in human. The function of LRRK2 has been most intensely studied in human cells because it has been identified as a risk factor for sporadic Parkinson's disease (Lill, 2016). LRRK2 has been detected in several intracellular locations and linked to a range of functions. It has been proposed to be associated with mitochondria where it interacts with DLP1 and regulates mitochondrial dynamics and function (Wang et al., 2012). On endosomal compartments, LRRK2 may associate with NAADP receptors and regulate lysosomal calcium homeostasis and autophagy (Gomez‐Suaga et al., 2012). In human macrophages, LRRK2 associates with the class III phosphatidylinositol‐3 kinase complex and Rubicon and inhibits the maturation of phagosomes containing *Mycobacterium tuberculosis* bacteria (Hartlova et al., 2018). Detailed studies will be necessary to establish if *Dictyostelium* LrrkA recapitulates some of the functions of human LRRK2 or whether Lrr kinases control different cellular functions in human and in *Dictyostelium* cells.

## 4 EXPERIMENTAL PROCEDURES

### 4.1 Strains and cell culture

*Dictyostelium* cells were grown at 21°C in HL5 medium (Froquet, Lelong, Marchetti, & Cosson, 2009). *Dictyostelium* cells used in this study were all derived from the DH1‐10 parental strain (Cornillon et al., 2000), referred to in this study as WT. The *kil2* KO and *kil1* KO strains were described previously (Leiba et al., 2017).

Bacterial strains were grown overnight in Luria–Bertani medium at 37°C. Bacteria used were uncapsulated *K. pneumoniae* KpGe (Lima et al., 2018), capsulated *K. pneumoniae* LM21 (Balestrino, Ghigo, Charbonnel, Haagensen, & Forestier, 2008), *P. aeruginosa* PT531 (Cosson et al., 2002), *E. coli* B/r, *B. subtilis* 36.1, and *M. luteus* (Benghezal et al., 2006). To generate fluorescent *K. pneumoniae* bacteria, the KpGe strain of *K. pneumoniae* was transfected with a plasmid constitutively expressing codon‐optimised yeast‐enhanced GFP (ye‐GFP) and conferring resistance to kanamycin. For this, ye‐GFP was amplified by PCR from pZA2 (addgene 97760; using as oligos: Oligo1: ACCGAATTCATTTTGACAGCTAGCTCAGTCCTAGGTATAATGCTAGCATTAAAGAGGAGAAATACTAGATGTCTAAAGGTGAAGAATTATTCACTGG and Oligo2: ATTAAGCTTTCATTTGTACAATTCATCCATACCATGGG). The PCR fragment (765 bp) was cloned into pZA2 (*Eco*RI/*Hind*III), thus switching the IPTG inducible promotor, pLac, to the strong synthetic constitutive promoter contained in Oligo1. A flagella‐less *B. subtilis* expressing mCherry (DK4214 Δhag *amyE*::P_*hyspank*_‐*mCherry spec*) was a kind gift of Professor D. Kearns (Indiana University, USA).

### 4.2 Screening for growth‐deficient *Dictyostelium* mutants

*Dictyostelium* mutants unable to grow in the presence of bacteria were isolated as described previously (Leiba et al., 2017). Briefly, *kil2* KO cells were mutagenised by restriction enzyme‐mediated insertion (REMI) of the pSC plasmid, a variant of pUCBsrΔBamHI (Adachi, Hasebe, Yoshinaga, Ohta, & Sutoh, 1994) with a modified polylinker (Figure S1e). Individual mutant cells were deposited in HL5‐containing 96‐well plates using a cell sorter, grown for 10 days, and then tested for their ability to grow efficiently on several bacteria: *M. luteus*, *B. subtilis*, *E. coli* B/r, *K. pneumoniae* KpGe, *K. pneumoniae* LM21, and *P. aeruginosa* PT531. Mutants that grew poorly on at least one of the tested bacteria were selected and further characterized.

A KO plasmid was constructed to replace a sequence in the *lrrkA* gene with a blasticidin‐resistance cassette, in WT, *kil1* KO, or *kil2* KO strains (Figure S1). Individual *lrrkA* KO clones were identified by PCR (Figure S1). At least two independent clones of each mutant were obtained and yielded identical results in this study.

### 4.3 Growth of *Dictyostelium* in the presence of bacteria

*Dictyostelium* cells were grown in the presence of bacteria as described previously (Froquet et al., 2009; Leiba et al., 2017). Briefly, 50 μl of an overnight bacterial culture was plated on 2 ml of Standard Medium (SM, for 1 l: 10 g peptone, 1 g yeast extract, 2.2 g KH2PO4, 1 g K2HPO4, 1 g MgSO4 : 7H2O, 10g glucose) agar in each well of a 24‐well plate. Then, 10, 100, 1,000, or 10,000 *Dictyostelium* cells were added on top of the bacterial lawn. Growth of *Dictyostelium* generated phagocytic plaques after 4–7 days of incubation at 21°C.

### 4.4 Intracellular killing of bacteria

To visualise phagocytosis and intracellular killing of individual bacteria, *K. pneumoniae* bacteria constitutively expressing a fluorescent GFP were mixed with *Dictyostelium* cells at a ratio of 3:1 in phosphate buffer (PB: 2 mM of Na_2_HPO_4_ and 14.7 mM of KH_2_PO_4_, pH 6.0) supplemented with 100 mM of sorbitol, deposited on an eight‐well slide (Ibidi μ‐slide 8 well Glass Bottom, 80827) for 10 min, and then imaged every 30 s for 2 hr with a videotime lapse (Nikon Eclipse T*i*2 equipped with a DS‐Qi2 camera) as described previously (Froquet et al., 2009; Leiba et al., 2017). At each time, one picture (phase contrast and GFP fluorescence) was taken in five successive focal planes (step size 3 μm) to image the whole‐cell volume. Four samples were analysed in parallel in this set‐up, generating paired series of measures. The Nikon NIS software (NIS Element AR 5.02.00) was used to extract images, and FiJi (v1.52j) was used to compile and analyse movies. Survival of at least 30 phagocytosed fluorescent bacteria was computed using the Kaplan–Meier estimator. In each experiment, the area under the survival curve was calculated, and a number between 1 (very rapid killing) and 7,500 (no killing) was obtained. To compare two conditions meaningfully (e.g., mutant vs. WT or not treated vs. folate treated), at least five independent experiments were performed and compared. Statistical comparisons were done with a paired Student's *t* test.

### 4.5 Sequence analysis

Domain searches were done with Interpro, TMHMM v.2.0, and SignalP v.4.1 servers. Searches for domains with a structure similar to LrrkA were done on the UniProt server using the following syntax: (ipr032675 OR ipr003591 OR ipr001611; ipr011009 OR ipr000719 OR ipr001245) NOT (keyword:transmembrane OR annotation:(type:transmem)).

### 4.6 Organisation and function of endosomal and lysosomal pathways

To perform immunofluorescence analysis, 10^6^ *Dictyostelium* cells were let to adhere to a glass coverslip for 30 min in HL5 medium. Cells were then fixed with 4% paraformaldehyde for 30 min, washed, permeabilised with methanol at −20°C for 2 min, and labelled with the indicated primary antibody in PBS containing 0.2% BSA for 1 hr. For this, antibodies against p80 (H161), p25 (H72; Ravanel et al., 2001), vacuolar H^+^‐ATPase (221‐35‐2), and rhesus (Benghezal et al., 2001) were used. Cells were then stained with fluorescent secondary antibodies for 1 hr and observed by LSM800 confocal microscopy (Carl Zeiss).

To determine the pH of phagosomes, we used pH‐sensitive bifluorescent beads as previously described (Sattler et al., 2013). Carboxylated silica beads (3 μm; Kisker Biotech; PSI‐3.0COOH) were coupled with both a green pH‐sensitive fluorescent probe (FITC) and a pH‐insensitive probe (Alexa 594 succinimidyl ester; Thermo Fisher A20004). They were mixed with *Dictyostelium* cells at a ratio of 5:1 in PB–sorbitol, deposited on an eight‐well slide (Ibidi μ‐slide 8 well Glass Bottom, 80827) for 10 min, and then imaged every 75 s for 3 hr with a videotime lapse (Nikon Eclipse T*i*2 equipped with a DS‐Qi2 camera) as described above.

To measure the proteolytic activity in phagosomes, we used proteolysis‐sensitive bifluorescent beads (Sattler et al., 2013). Three‐micrometer carboxylated silica beads (Kisker Biotech; PSI‐3.0COOH) were coupled with both a proteolysis‐sensitive probe (DQ™ Green BSA; Thermo Fisher D12050) and a proteolysis‐insensitive probe (Alexa 594 succinimidyl ester; Thermo Fisher A20004).

The activity of lysosomal glycosidases in cells and supernatants was measured as previously described (Le Coadic et al., 2013) using a colorimetric assay. Briefly, cells were grown to a density of 2 × 10^6^ cells per ml. Cells and medium were separated by centrifugation (1,500 *g*, 2 min), and the glycosidase activity (*N*‐acetyl‐β‐glucosaminidase and α‐mannosidase) revealed using chromogenic substrates (*p*‐nitrophenyl‐*N*‐acetyl‐β‐d‐glucosamide and *p*‐nitrophenyl‐α‐d‐mannopyranoside, respectively). Release of *para*‐nitrophenol upon glycolysis was measured by spectrophotometry (405 nm).

### 4.7 Lysozyme activity

To measure lysozyme activity, 2.5 × 10^8^ *Dictyostelium* cells were washed twice in PB buffer and lysed with 600 μl of lysis buffer (50 mM of sodium PB pH 3, 0.5% Triton X‐100, 20 μg ml^−1^ of leupeptin, 10 μg ml^−1^ of aprotinin, and 18 μg ml^−1^ of phenylmethylsulfonyl fluoride [PMSF]). The suspension was centrifuged (30,000 *g* during 10 min at 4°C), and the supernatant was collected and diluted in lysis buffer. Muramidase activity was assessed by mixing in a microtitre plate 100 μl of cell lysate with 100 μl of heat‐killed *Micrococcus lysodeikticus* (Sigma) suspended in 50 mM of PB (pH 3) to a final optical density at 450 nm of 0.5. The decrease in turbidity (optical density at 450 nm) after 2 hr of incubation at 21°C was measured with a spectrophotometer plate reader and used to determine the muramidase activity of *lrrk*A KO cells relative to their WT counterparts.

### 4.8 Autophosphorylation of LrrkA

#### 4.8.1 Construction of vectors for expression of tagged LrrkA proteins

A DNA fragment corresponding to the entire open reading frame of *lrrkA* was amplified by PCR using cDNAs as template. The amplified fragment was subcloned into pCR‐Blunt II‐TOPO (Invitrogen). The coding sequence was then subcloned in pLD1ΔBX‐myc (Kawata et al., 2015) to yield an expression vector encoding LrrkA tagged with the myc epitope.

For point mutation of the conserved ATP‐binding site (Hanks & Hunter, 1995; lysine residue at 878) of the LrrkA kinase domain, an inverse PCR was performed with KOD plus Mutagenesis Kit (TOYOBO, Osaka, Japan) according to the manufacturer's instruction. The LrrkA (K878A) fragment was purified and ligated as above into pLD1ΔBX‐myc. The resultant vectors were transformed into *lrrkA* KO cells to produce LrrkA–Myc and LrrkA (K878A)–Myc, a kinase‐dead form of LrrkA.

Immunoprecipitation of Myc‐tagged LrrkA was performed as previously described (Araki et al., 1998): Cells (6.0 × 10^8^) were washed twice in KK2 buffer (16.5 mM of KH_2_PO_4_ and 3.8 mM of K_2_HPO_4_, pH 6.2), suspended in KK2 buffer at a density of 2 × 10^7^ cells per ml, and shaken for 4 hr at 150 r.p.m., followed by addition of cAMP (Tokyo chemical industry, Tokyo, Japan) to achieve a final concentration of 5 mM, and further shaken for 15 min. Cells were harvested and lysed in 6 ml of mNP40 lysis buffer (50 mM of Tris–HCl (pH 8.0), 150 ml of NaCl, 1.0% (v/v) Nonidet P‐40, 50 mM of NaF, 2 mM of EDTA (pH 8.0), 2 mM of Na pyrophosphate, 2 mM of benzamidine, 1 μg/ml of pepstatin, 1 mM of PMSF, and complete EDTA‐free protease inhibitor mixture [Roche Diagnostics]) for 10 min on ice. After preclearing by centrifugation, the supernatant was incubated with anti‐Myc antibody for 2–3 hr at 4°C with gentle rocking, followed by additional 2–3 hr of incubation with Dynabeads Protein‐G (Thermo Fisher Scientific). Alternatively, the supernatant was directly incubated with anti‐Myc tag magnetic beads (M047‐9, MBL International Co.) for 2 hr. Beads were washed four times in mNP40 buffer, and proteins were eluted by boiling in SDS gel sample buffer and then subjected to Western blot analysis.

The primary antibodies used in the experiments were anti‐phosphotyrosine antibody 4G10 (Merck Millipore) for general phosphotyrosine modification, anti‐phosphoserine antibody A8G9 (Abnova), and anti‐Myc antibody 9E10 (Wako Pure Chemical, Osaka, Japan) for Myc‐tagged proteins. Alkaline phosphatase‐conjugated anti‐mouse IgG (H + L) antibody (S372B, Promega) or anti‐rabbit IgG (Fc) antibody (S373B, Promega) was used as the secondary antibody for Western blot analysis. Proteins were detected using the Western Blue® Stabilized Substrate for Alkaline Phosphatase (S3841, Promega).

## Supporting information


**Figure S1**. Isolation and generation of *lrrkA* KO cells. (A) Schematic representation of the *lrrkA* insertional mutant obtained by REMI mutagenesis, with the mutagenic pSC plasmid inserted 1'782 nucleotides (nt) Bodinier et al. Supplementary figures 2 after the start codon of *lrrkA*. The site of insertion was identified by digestion of genomic DNA with ClaI, which allowed the recovery of the mutagenic plasmid with the genomic flanking regions of *lrrkA*. (B) Schematic representation of the *lrrkA* gene in WT and in KO cells. To create *lrrkA* KO cells, we deleted 880 nt of the genomic sequence, 1'048 nt downstream of the *lrrkA* start codon and replaced this portion with a blasticidin resistance cassette by homologous recombination. Arrows indicate the positions of the oligonucleotides used to identify KO cells. (C‐D) Identification of *lrrkA* KO cells was done by PCR using distinct pairs of oligonucleotides to verify both loss and gain of signal. Three independent *lrrkA* KO clones were identified. (E) Structure of the pSC plasmid. The overall structure of the plasmid is indicated, as well as the sequence of the cloning site.
**Figure S2**. Detailed structure of all *Dictyostelium* LRR kinase proteins. The main functional domains present in each protein are indicated. Note that Roco7 is strictly speaking not an LRR kinase, since it lacks LRRs. The structure of the human LRRK1 and 2 is also shown for comparison. Domains were drawn using “Illustrator for Biological Sequences” (http://ibs.biocuckoo.org).
**Figure S3**. LrrkA is capable of self‐phosphorylation on a serine residue. Cells expressing either LrrkA‐Myc (WT) or LrrkA(K877A)‐Myc were harvested and starved in KK2 buffer for 4 h. After starvation, cAMP was added and incubated further 15 min. Myc‐tagged LrrkA was immunoprecipitated with the 9E10 anti‐myc antibody, and the precipitated samples were subjected to Western blot analysis. The blot was probed with anti‐phosphoserine antibody A8G9 (upper row; pSer), anti‐phosphotyrosine antibody 4G10 (middle row; pTyr), or 9E10 (lower row; c‐Myc).
**Figure S4**. Intracellular killing of *B. subtilis* is unaffected in *lrrkA* KO cells. *Dictyostelium* cells were incubated with mCherry‐expressing *B. subtilis (Bs)* in PB‐sorbitol for 2 h. Cells were observed by phase contrast and fluorescence microscopy, and the ingestion and intracellular killing of *Bs* monitored. (A) The probability of bacterial survival following ingestion is represented as a Kaplan‐ Meyer estimator for one experiment in WT cells (n=91 ingested bacteria) (white squares) and *lrrkA* KO cells (n=76) (black squares). (B) For three independent experiments, the survival of bacteria was determined by measuring the area under the survival curve from 0 to 75 min. Intracellular killing was not different in WT and *lrrkA* KO cells (Wilcoxon matched‐pairs rank test, N=3, p=0.75)
