# Supplementary material for: LrrkA, a kinase with leucine‐rich repeats, links folate sensing with Kil2 activity and intracellular killing
Source: Cell Microbiol. 2019 Nov 7;22(1):e13129. doi: 10.1111/cmi.13129 (PMC7003747; doi:10.1111/cmi.13129)
Supplement: Supplementary file 1 — Figure S1. Isolation and generation of lrrkA KO cells. (A) Schematic representation of the lrrkA insertional mutant obtained by REMI mutagenesis, with the mutagenic pSC plasmid inserted 1'782 nucleotides (nt) Bodinier et al. Supplementary figures 2 after the start codon of lrrkA. The site of insertion was identified by digestion of genomic DNA with ClaI, which allowed the recovery of the mutagenic plasmid with the genomic flanking regions of lrrkA. (B) Schematic representation of the lrrkA gene in WT and in KO cells. To create lrrkA KO cells, we deleted 880 nt of the genomic sequence, 1'048 nt downstream of the lrrkA start codon and replaced this portion with a blasticidin resistance cassette by homologous recombination. Arrows indicate the positions of the oligonucleotides used to identify KO cells. (C‐D) Identification of lrrkA KO cells was done by PCR using distinct pairs of oligonucleotides to verify both loss and gain of signal. Three independent lrrkA KO clones were identified. (E) Structure of the pSC plasmid. The overall structure of the plasmid is indicated, as well as the sequence of the cloning site. Figure S2. Detailed structure of all Dictyostelium LRR kinase proteins. The main functional domains present in each protein are indicated. Note that Roco7 is strictly speaking not an LRR kinase, since it lacks LRRs. The structure of the human LRRK1 and 2 is also shown for comparison. Domains were drawn using “Illustrator for Biological Sequences” (http://ibs.biocuckoo.org). Figure S3. LrrkA is capable of self‐phosphorylation on a serine residue. Cells expressing either LrrkA‐Myc (WT) or LrrkA(K877A)‐Myc were harvested and starved in KK2 buffer for 4 h. After starvation, cAMP was added and incubated further 15 min. Myc‐tagged LrrkA was immunoprecipitated with the 9E10 anti‐myc antibody, and the precipitated samples were subjected to Western blot analysis. The blot was probed with anti‐phosphoserine antibody A8G9 (upper row; pSer), anti‐phosphotyrosine antibody [file CMI-22-e13129-s001.pdf]

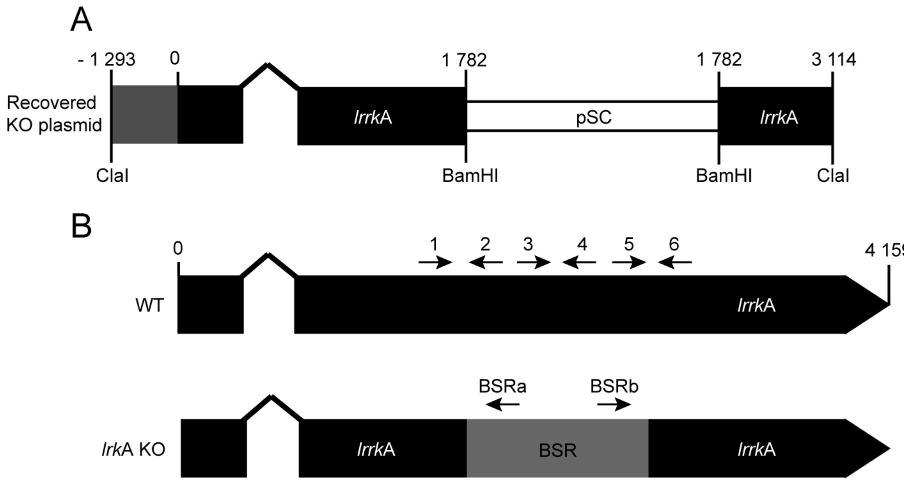

C

Oligos	Sequence
1	GATGCTTCATGAAGAAGCAGAG
2	GATTGGCTGATAGATCAAGATCACG
3	CGTGATCTTGATCTATCAGCCAATC
4	ATTGGTGGAAGTGCAGGGTTAACA
5	GAGGAACAGAAGTAGCAGTGAAAATGC
6	TTGGTGACCCAGTTTGTTGTAGTTG
BSRa	TCAAAAAGATAAAGCTGACCCGAAAGC
BSRb	TTCAAATAATAATTAACCAACCCAAG

	Oligos pair	Size WT (bp)	Size KO (bp)
Loss of signal	1+2	590	0
	3+4	515	0
	5+6	1152	0
Gain of signal	1+BSRa	0	638
	BSRb+6	0	1187

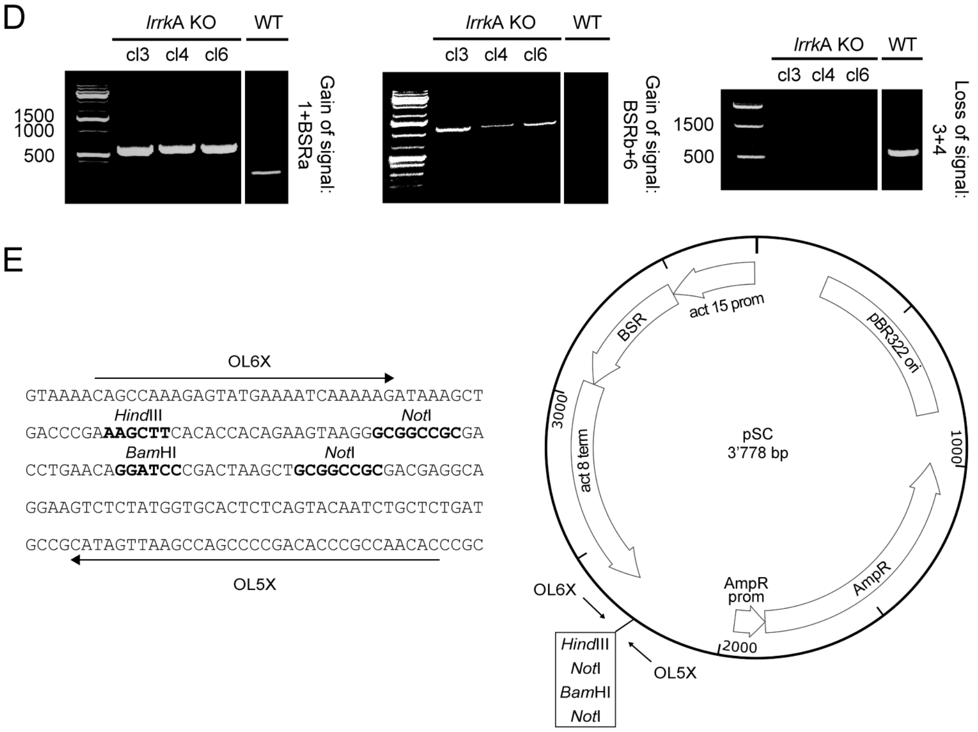

Figure S1. Isolation and generation of *IrrkA* KO cells.

(A) Schematic representation of the *IrrkA* insertional mutant obtained by REMI mutagenesis, with the mutagenic pSC plasmid inserted 1'782 nucleotides (nt)

after the start codon of *lrrkA*. The site of insertion was identified by digestion of genomic DNA with ClaI, which allowed the recovery of the mutagenic plasmid with the genomic flanking regions of *lrrkA*. (B) Schematic representation of the *lrrkA* gene in WT and in KO cells. To create *lrrkA* KO cells, we deleted 880 nt of the genomic sequence, 1’048 nt downstream of the *lrrkA* start codon and replaced this portion with a blasticidin resistance cassette by homologous recombination. Arrows indicate the positions of the oligonucleotides used to identify KO cells. (C-D) Identification of *lrrkA* KO cells was done by PCR using distinct pairs of oligonucleotides to verify both loss and gain of signal. Three independent *lrrkA* KO clones were identified. (E) Structure of the pSC plasmid. The overall structure of the plasmid is indicated, as well as the sequence of the cloning site.

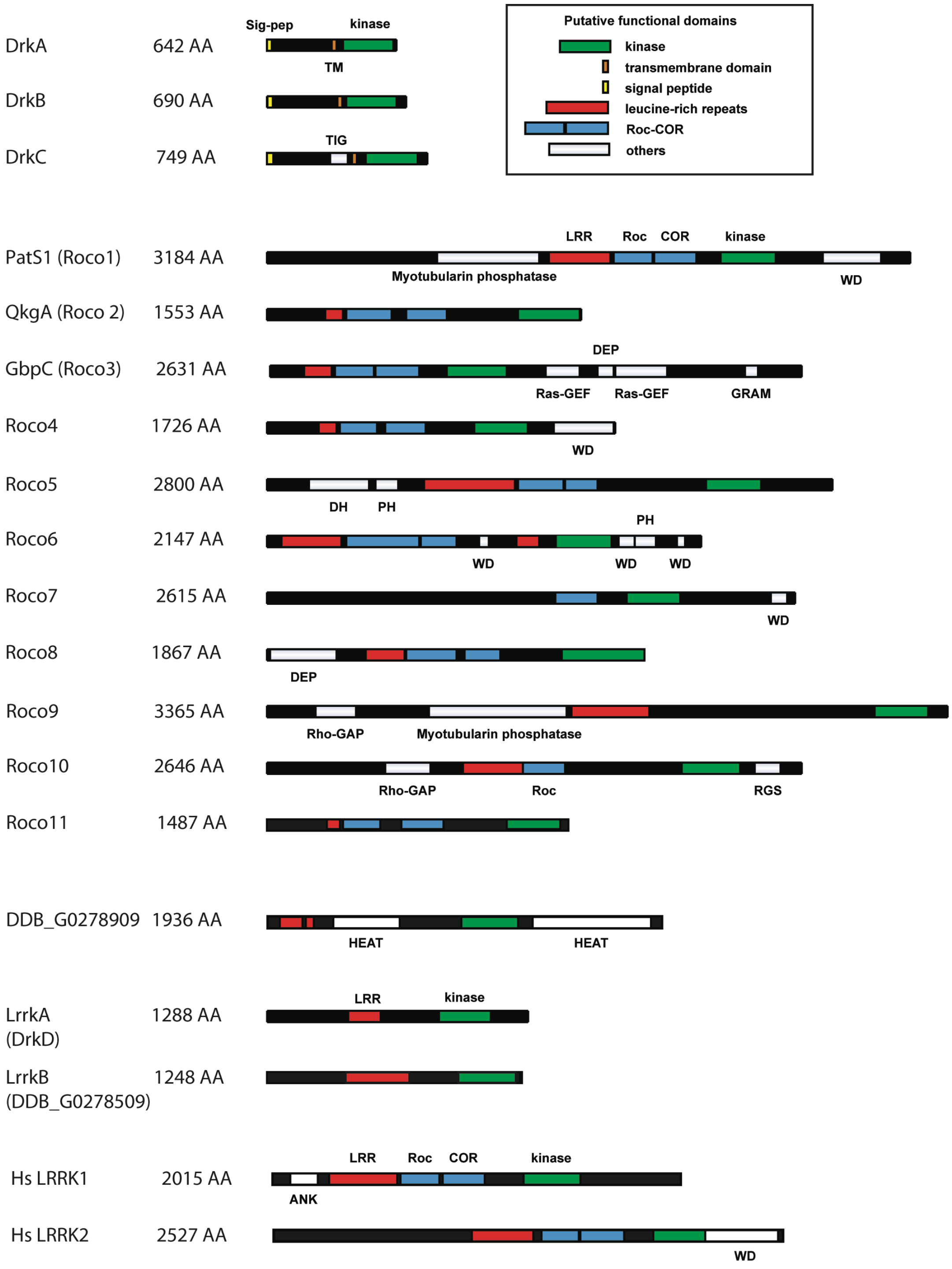

Figure S2. Detailed structure of all *Dictyostelium* LRR kinase proteins. The main functional domains present in each protein are indicated. Note that Roco7 is strictly speaking not an LRR kinase, since it lacks LRRs. The structure of the human LRRK1 and 2 is also shown for comparison. Domains were drawn using "Illustrator for Biological Sequences" (http://ibs.biocuckoo.org).

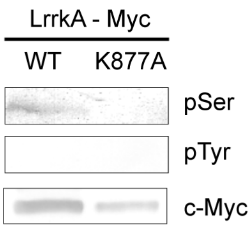

Figure S3. LrrkA is capable of self-phosphorylation on a serine residue.

Cells expressing either LrrkA-Myc (WT) or LrrkA(K877A)-Myc were harvested and starved in KK2 buffer for 4 h. After starvation, cAMP was added and incubated further 15 min. Myc-tagged LrrkA was immunoprecipitated with the 9E10 anti-myc antibody, and the precipitated samples were subjected to Western blot analysis. The blot was probed with anti-phosphoserine antibody A8G9 (upper row; pSer), anti-phosphotyrosine antibody 4G10 (middle row; pTyr), or 9E10 (lower row; c-Myc).

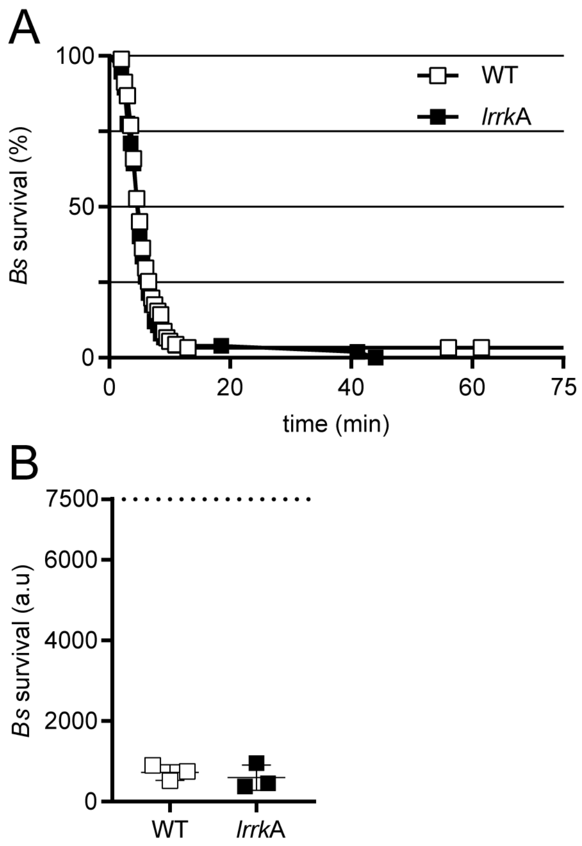

Figure S4: Intracellular killing of *B. subtilis* is unaffected in *lrrkA* KO cells. *Dictyostelium* cells were incubated with mCherry-expressing *B. subtilis (Bs)* in PB-sorbitol for 2 h. Cells were observed by phase contrast and fluorescence microscopy, and the ingestion and intracellular killing of *Bs* monitored. (A) The probability of bacterial survival following ingestion is represented as a Kaplan-Meyer estimator for one experiment in WT cells (n=91 ingested bacteria) (white squares) and *lrrkA* KO cells (n=76) (black squares). (B) For three independent experiments, the survival of bacteria was determined by measuring the area under the survival curve from 0 to 75 min. Intracellular killing was not different in WT and *lrrkA* KO cells (Wilcoxon matched-pairs rank test, N=3, p=0.75)
